# *In Silico* and* In Vitro* Analysis of the 4,4′,4′′-((1,3,5-Triazine-2,4,6-triyl)tris(azanediyl))triphenol), an Antioxidant Agent with a Possible Anti-Inflammatory Function

**DOI:** 10.1155/2019/9165648

**Published:** 2019-05-22

**Authors:** Ricardo I. Castro, Felipe Valenzuela-Riffo, Luis Morales-Quintana

**Affiliations:** ^1^Multidisciplinary Agroindustry Research Laboratory, Instituto de Ciencias Químicas Aplicadas, Universidad Autónoma de Chile, Talca, Chile; ^2^Instituto de Ciencias Biológicas, Universidad de Talca, Talca, Chile; ^3^Multidisciplinary Agroindustry Research Laboratory, Instituto de Ciencias Biomédicas, Facultad de Ingeniería, Universidad Autónoma de Chile, Talca, Chile

## Abstract

Inflammation is a consequence of an array of biological reactions that occur in response to pain sensation, local injury, and cell damage. A large number of studies have demonstrated that quercetin and other flavonoids show anti-inflammatory effects; thus, in the present work, we evaluated a triazine-phenol derivative (TP derivative) compound as a possible drug candidate with anti-inflammatory activity. This compound was studied as a possible anti-inflammatory drug using synthesis and characterization by Fourier transform infrared spectroscopy (FTIR), thermogravimetric analysis (TGA), and mass spectrometry (MS). The derivative of melamine was evaluated for its antioxidant activity and exhibited good DPPH and FRAP antioxidant activity. Additionally, we evaluated the putative effect of the molecule on the COX-2 enzyme through molecular dynamic simulation (MDS), and the result suggested that the TP derivative is a potential anti-inflammatory agent that can interact with the COX-2 enzyme because of the high number of protein-ligand interactions observed with MDS. Finally, the study of theoretical physicochemical properties, the observation of low toxicity (hemolysis assay), and the evaluation of oral bioavailability of the TP derivative showed that it is a possible anti-inflammatory drug candidate.

## 1. Introduction

Inflammation is a consequence of an array of reactions at the biological level that occur in response to pain sensation, local injury, and cell damage [1, 2]. Many factors have been reported that may induce inflammation, including recovery from injury, antigen-antibody reactions, and defense against pathogenic organisms [3]. Additionally, there are numerous anti-inflammatory mechanisms that can explain these processes. One of the most important mechanisms is the inhibition of cyclooxygenase (COX). It has been discovered that two different COX enzymes exist, COX-1 and COX-2. Cyclooxygenase-1 (COX-1) is involved mainly in the functions of the lining of the stomach, the kidney, and platelets. Cyclooxygenase-2 (COX-2), which is cytokine-inducible, is expressed in inflammatory cells [4, 5].

Currently, nonsteroidal drugs and other anti-inflammatory drugs are used to relieve inflammation. However, the use of these drugs is limited because of their side effects, such as gastric ulcers, renal damage, bronchospasm, and cardiac abnormalities [2]. Natural products have been reported to be beneficial to health because of their possible effects on the prevention of diseases such as cardiovascular disease, different types of cancer, and inflammatory disease. Additionally, natural products have been an important source for the design of drugs targeting various pathologies, including the design of anti-inflammatory agents [6, 7]. Thus, new similar compounds obtained from natural products such as phenolic acids and flavonoids represent alternative sources of new drugs because of their antioxidant properties [8], biological functions [9], and anti-inflammatory properties [10, 11].

In contrast, 1,3,5-triazine derivatives are widely used as herbicides [12], drugs [13], or polymers [14], such as melamine-formaldehyde, which has excellent thermal and electrical properties. Similarly, some time ago de Hoog et al. (2002) [15] showed the synthesis reaction of a TP-derived compound. In the present study, we evaluated the anti-inflammatory properties and antioxidant properties of this compound. Thus, the aims of this research were to synthesize and characterize a triazine-derived compound with phenolic group substitutions. To that end, we characterized the* in vitro* antioxidant activity and toxicity. Additionally, using* in silico* tools (molecular docking and molecular dynamics simulations), we described the protein-ligand interactions involved in the binding mode of our proposed compound and the active site of the putative target protein, COX-2 enzyme, an enzyme with a crucial role in the inflammation response.

## 2. Materials and Methods

### 2.1. Synthesis of the 4,4′,4′′-((1,3,5-Triazine-2,4,6-triyl)tris(azanediyl))triphenol) Compound

**Scheme 1:**
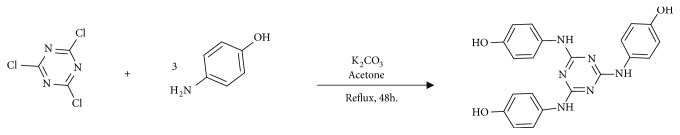
Synthesis of 4,4′,4′′-((1,3,5-triazine-2,4,6-triyl)tris(azanediyl))triphenol).

The synthesis of the derivative of triazine (Scheme 1) was accomplished by adding 2.50 g (13.55 mmol) of 2,4,6-trichloro-1,3,5-triazine (Sigma-Aldrich) dissolved in 50 mL of acetone. Then, 2.81 g (20.30 mmol) of potassium carbonate was added to the flask and cooled to 0°C, and 4.44 g (40.65 mmol) of 4-aminophenol was added. Next, the suspension mixture was warmed to room temperature and refluxed for 48 h. The solid obtained was filtered under reduced pressure and washed with Milli-Q water (3 × 50 mL) to remove the salt KCl formed [15].

### 2.2. Characterization of the Compound

A Fourier transform infrared (FTIR) spectrophotometer (Nicolet Nexus 470) was employed to obtain the spectra of the MG resin. The spectra were collected using the Smart Orbit Attenuated Total Reflectance (ATR) accessory. Thermogravimetric analysis (TGA) was conducted under a nitrogen atmosphere at a heating rate of 10°C min^−1^ on a TGA Q500 (TA Instruments). A mass spectrometer (Q-Tof Micromass UK) with a constant nebulizer temperature of 393 K was used. The values presented are the average mass and correspond to the [M+H] ion.

### 2.3. Red Blood Cell Lysis Assay

The hemolysis assay was performed according to the method of Duncan et al. (2005) [17]. In short, the red blood cells (RBCs) were washed with PBS (Phosphate Buffered Saline) at 2%, after which the cells were incubated at 30°C in a saline solution at a final concentration of 0.25 mg mL^−1^. After incubation for 2 h, the samples were centrifuged at 2000 rpm for 10 min, and the absorbance of the supernatant at 550 nm was measured. Hemolysis was expressed as the percentage of released hemoglobin. A solution of RBCs incubated with Triton X-100 (0.2% V/V) was used as a control (100% of hemoglobin released). Additionally, morphological changes in the RBCs were determined by optical microscopy.

### 2.4. Ferric Reducing Antioxidant Power (FRAP) Assay

The FRAP assay was conducted according to Castro et al., (2018) [18] using the reagent 2,4,6-tris and (2-pyridyl)-s-triazine dissolved in 40 mM of HCl, 25 mL of 300 mM acetate buffer at pH 3.6, and 2.5 mL of 20 mM FeCl_3_ ∗ 6 H_2_O. All samples were read at 593 nm using a spectrophotometer (Thermo Spectronic GENESYS 10UV). The percentage of Fe^3+^ scavenging (reduction to Fe^2+^) was calculated by comparison with the standard curve (per g of compound).

### 2.5. (1,1-Diphenyl-2-picrylhydrazyl) Radical Scavenging Method (DPPH)

The DPPH assay was conducted according to Forero et al. (2014) [19]. The absorbance was measured at 515 nm using a spectrophotometer (Thermo Spectronic GENESYS 10UV). The percentage of the half-maximal inhibitory concentration (IC_50_, mg/mL) was calculated by linear regression analysis of the mean of three determinations.

### 2.6. Protein-Ligand Interactions

Docking studies were performed to predict the putative binding interactions of the COX-2 protein (PDB code: 3LN0) with the TP derivative ligand and quercetin (PubChem CID: 5280343), which was employed as a positive control. The two ligands were optimized with the SCHRÖDINGER suite with the OPLS v2005 force field, specifically with the LigPrep [20] and Epik [21] tools. After ligand optimization, the protein geometry was optimized with the OPLS v2005 force field [22]. The docking experiments were performed using the extra precision mode in the GLIDE program [23], a suite of SCHRÖDINGER. Five independent docking runs were conducted for each ligand, and 25 conformers were obtained in each case.

The molecular dynamic simulations (MDS) of the ligands inside the recognition site of the COX-2 enzyme were studied using Desmond Package of SCHRÖDINGER [24] suite with OPLS v2005 force field [25]. The initial coordinates for the simulations were taken from the docking experiments. A crystal structure of COX-2 was embedded into a preequilibrated SPC model of water molecules, and NaCl was added to neutralize the system. All MDS were performed at constant temperature (300 K) and pressure (1.01325 bar) in an isothermal-isobaric ensemble. The MDS were performed with the protein secondary structure of COX-2 having a spring constant of 0.5 kcal mol^−1^ Å^−2^ during 100 ns. Data were collected every 50 ps during the trajectory. Finally, the two MDS were analyzed by a simulation interaction diagram in the SCHÖDINGER suite.

## 3. Result and Discussion

### 3.1. Characterization of (4,4′,4′′-((1,3,5-Triazine-2,4,6-triyl)tris(azanediyl))triphenol)

First, we characterized the TP compound, and in short, we found the following: white powder; yield=84%. The FTIR main signals were found at 3390, 3350, 1625, 1600, 1582, 1010, and 808 cm^−1^; the signals at 3390-3350 cm^−1^ are characteristic of residual amine groups and correspond to the symmetric vibration as well as the asymmetric stretching vibration of N-H, suggesting the formation of the derivative. Additionally, the spectra show characteristic absorption bands at 1625 and 1519 cm^−1^, which were attributed to N-H bending and C-N vibrations, respectively. Other important signals present in the molecule were in the region of 1010 and 808 cm^−1^, which was assigned to the NH bending (ring deformation) and the CNC + NCN bending (ring out of the deformation plane) of the triazine group (Figure S1).

Thermogravimetric analysis (TGA) was used to investigate the formation of the TP derivative, and the results showed that the initial weight loss was attributed to dehydration. The second weight loss at the temperatures between 100 and 230°C was attributed to the release of CO and CO_2_ from the groups containing the most labile oxygen molecules (phenol group) [26]. The weight loss at high temperatures was attributed to the decomposition of the derivative formed (Figure S2). Finally, the mass spectrometry analysis showed that ESI-MS (+): [M + H]^+^ 403 m/z and [M + K]^+^ 440,7 m/z (Figure S3).

### 3.2. Interaction TP Derivate with Red Blood Cells (RBCs)

**Figure 1:**
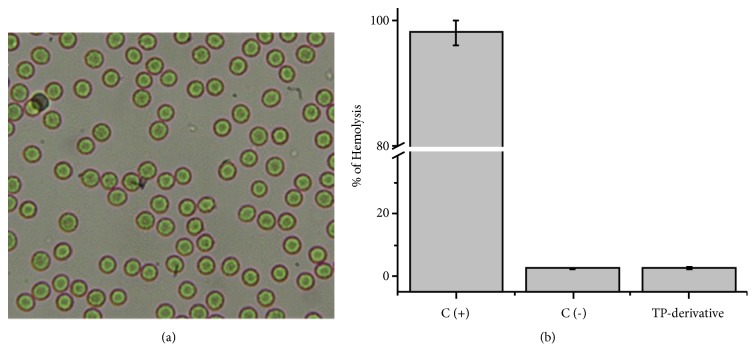
*Results of RBC hemolysis assay.* (a) Optical microscopy of washed RBCs after incubation with the TP derivative (TP derivative) (0.05 mg/mL). Scale bar: 20 mm. (b) Percent hemolysis of washed RBCs obtained from the interaction with the derivative of melamine compared to the positive control 0.2% Triton X-100 (100% hemolysis) and negative control (PBS) n=3.

Figure 1 shows the results of the hemolysis of RBCs, expressing the percentage of released hemoglobin relative to the positive control (Triton X-100, 0.2%, v/v). The results demonstrated that the TP derivative at a final concentration of 0.05 mg/mL induced a low percentage of hemolysis (7%). The analysis also shows that the derivative of triazine did not result in significant intense agglutination of RBCs. This demonstrates the low percentage toxicity of the derivative because any effects, such as aggregation, crenation, and hemolysis, on RBCs induced by external agents suggest incompatibility [27]. Thus, the TP derivative is proposed to be a potential drug.

### 3.3. DPPH, FRAP, and Antioxidant Structure-Activity Assays and the Relationship with the Number of Hydroxyl Groups

The antioxidant capacity of the TP derivative compound was determined by the free radical scavenging assay (DPPH) and the ferric reducing antioxidant power (FRAP) assay. The results showed an IC_50_ (mg/mL) of 4.90 ± 0.16 for the compound, while the IC_50_ was 1.48 ± 0.1 for quercetin. With respect to the capacity to reduce Fe^3+^ to Fe^2+^, the TP derivative compound showed a higher capacity with 9561.1 ± 77.8 (*μ*mol Fe^2+^/g) than quercetin with 11162.5 ± 94.2 (*μ*mol Fe^2+^/g), which is in accordance with previously published results capacity of quercetin in this assay [28]. Additionally, the antioxidant capacity of the TP derivative and quercetin (as a positive control) were compared using two different methods (FRAP and DPPH) (Table 1). The results showed that regardless of the method used, both compounds have a high antioxidant capacity (Table 1). However, depending on the method, there is a difference in which of the two is better; in the FRAP assay, the compound with the best antioxidant capacity was quercetin, and in the DPPH assay, the compound with the best antioxidant capacity was the TP derivative (Table 1). This antioxidant capacity is possible because both compounds have similar chemical compositions, mainly regarding the phenolic rings present in the structures of quercetin, as well as flavonoids in general, and the TP derivative [29, 30].

Another structural characteristic is the number of -OH substitutions, which is an important property of many natural products, such as flavones, that is related to antioxidant capacity. For example, in the evaluation of the ORAC ROO° activity of three different characteristic compounds with high antioxidant capacity (quercetin, kaempferol, and myricetin), there was a relationship between the antioxidant capacity and the number of -OH moieties, and the following order of antioxidant capacity was obtained: kaempferol < quercetin < myricetin. Quercetin contains 5 hydroxyl groups, while the TP derivative compound only has 3 hydroxyl groups [31] (Scheme 1).

### 3.4. *In Silico* Protein-Ligand Interaction

COXs [30], also known as prostaglandin G/H synthases, play a crucial role in inflammation and are targets of common nonsteroidal anti-inflammatory drugs [32]. There are two main COX enzymes, COX-1 and COX-2. In particular, different authors have suggested a beneficial action of COX-2 in inflammation [33–37]. Thus, we used the COX-2 structure to evaluate the molecular mechanism of the triazine derivative compound as an anti-inflammatory drug. The crystal structure of COX-2 was evaluated using the TP derivative and quercetin as the ligands. First, a molecular docking simulation was used to bring the molecules together and to include the ligands in the active site of the COX-2 enzyme; negative energies were obtained for the two ligands tested, indicating a probable protein-ligand interaction. The best interaction was obtained between COX-2 and the TP derivate as substrate with −54.2 kcal mol^−1^, while quercetin showed a value of −43.6 kcal mol^−1^ (Table 2).

**Figure 2:**
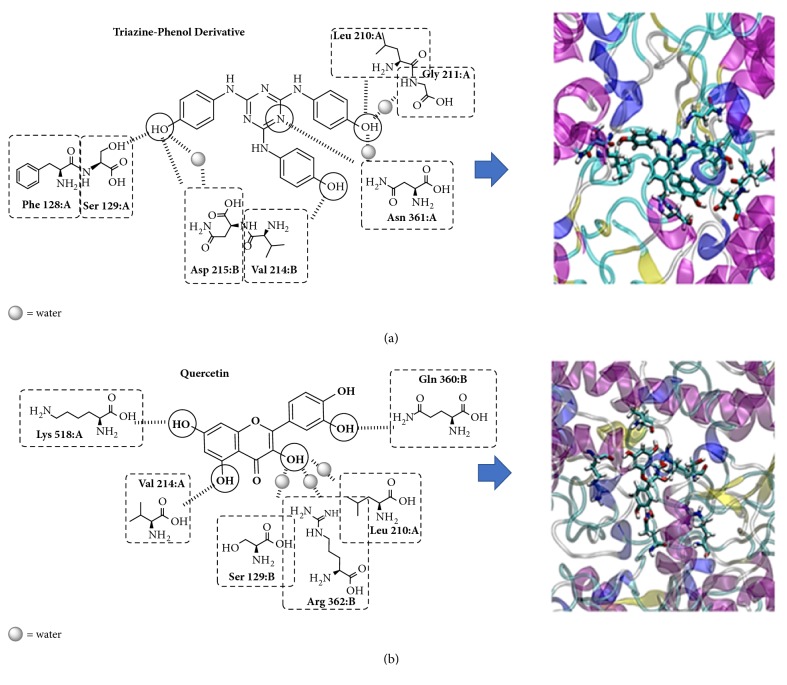
Residues involved in the protein-ligand interaction of each ligand and the COX-2 enzyme.

**Figure 3:**
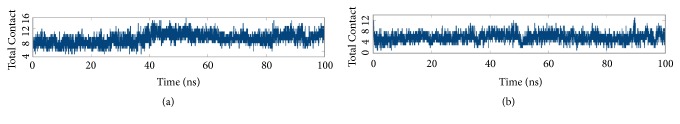
A timeline representation of the interactions and total contacts (H-bonds, hydrophobic interactions, ionic interactions, and water bridges) obtained during the molecular dynamics simulations. The panels show the total number of specific contacts the protein made with the ligand over the course of the simulation. (a) corresponds to the TP derivative, and (b) corresponds to quercetin.

MDS was performed with each ligand using the best protein-ligand complex obtained from the MM-GBSA and docking studies. The analysis of the molecular dynamics simulations showed that the two ligands could interact with the COX-2 enzyme with stable RMSD values of the ligands with the protein (Figure S4); however, a lower RMSD of 1.6 Å was obtained for the TP derivative than for quercetin, which showed an RMSD value of approximately 3.5 Å (Figure S4). The protein-ligand interactions can be monitored throughout the simulation, and we showed different residues that directly or indirectly interact with each ligand.  Table 3 shows the hydrogen bonding (H-bond) frequency between the COX-2 residues and each ligand, indicating that 11 residues play the most important roles in the COX-2_ligand complex. Ten of these residues are more persistent in H-bond formation and one of these residues is important to maintain the structure of the pocket. Interestingly, all eleven residues do not participate in the interactions with both ligands; only six residues participate in the COX-2_quercetin interaction, while nine residues participate in the COX-2_TP derivative interaction (Table 3). Additionally, Figure 2 shows the interaction geometries of the TP derivative and quercetin; the residues of chain A and B participate in the COX-2_ligand interaction, and there are small differences between the two putative drugs. However, these residues that form H-bonds with the ligands are not the only residues that interact with the ligands; in fact, the protein-ligand interaction can form “contacts”, and these “contacts” are categorized into four types: hydrogen bonds (shown in Table 3), hydrophobic interactions, ionic interactions, and water bridges (Figure S5). Figure S5 shows the other types of interactions present in each complex, and the TP derivative ligand has more interactions than quercetin and was interacting with COX-2 for a large percentage of time (Figure S5). The value of 0.8 of the Phe128 residue (chain A) in Figure S5 in panel A indicates that the hydrophobic interaction between this residue and the ligand was maintained for 80% of the simulation time, while values over 1.0 indicate that the protein residue may make multiple contacts of the same subtype with the ligand, such as the residue Asp 215 of chain B in Figure S5, panel A. Finally, all the interactions of the residues separately shown in both Figure S5 and Table 3 indicate that the TP derivative ligand, with approximately 8 to 10 average interactions, has a greater number of contacts during the MDS than quercetin, which showed approximately 6 or 7 interactions per time step during the MDS (Figure 3).

### 3.5. Structure-Activity and Anti-Inflammatory Correlation

**Figure 4:**
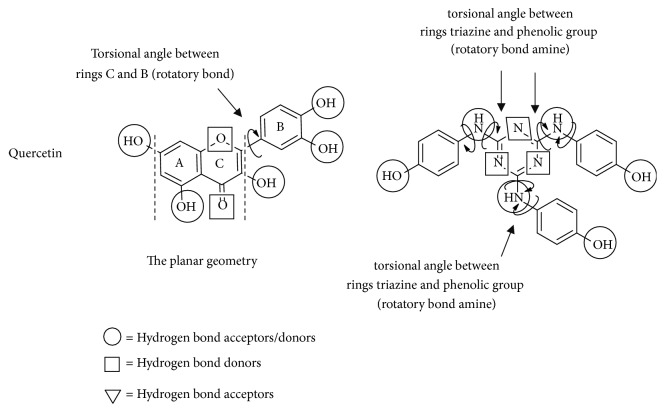
Structure and torsion angle of the main bond and acceptor/donor groups.

The relationships between drug structure and drug activity were determined by MDS, and the results show that the activity of quercetin and the TP derivative at the active site of COX-2 is mainly mediated by the hydroxyl groups present in both structures (Figure 4). In the case of quercetin, there is a strong intramolecular hydrogen bond between the hydroxyl of C5 (5-OH group) and the carbonyl oxygen [38] that does not favor the anti-inflammatory activity. However, although the nitrogen or the nitrogen group atoms present in the structure of the heterocycle derivative of pyridine could increase interactions with the active site (e.g., the drug etoricoxib), the MDS showed that the heterocyclic structure present in the TP derivative did not favor the interaction. The reduced interaction between the TP derivative and COX-2 can be explained by the steric hindrance produced by the interaction of the center of molecule with the active site by the three aromatic rings present in the border of the structure. Another possible point of interaction is the amine group present between the phenolic group and the triazine nucleus. These groups do not contribute in the interaction with the active site because of the steric hindrance of the aromatic ring (Figure 4).

### 3.6. Relationships between Conformational Analysis and Anti-Inflammatory Activity

The conformational preferences between the interaction site and the ligand were studied to compare the conformational structure of both ligand molecules. The results showed that quercetin has a minor point interaction with COX-2, mainly because there is only 1 rotatory bond (Table 3). Specifically, this rotational bond is found between C2 and C1', which is formed from orbital C_sp2_-C_sp3_ that allows the bonds to rotate freely. The heavy electron density in the *σ* bond between the two explains the minor interaction of the ligand residue (Figure 4). This rotational bond allows the OH group between C3' and C4' in the quercetin molecule to flip (Figure 4). With respect to the TP derivative molecule, 6 possible conformations were observed because this molecule has 6 rotatory bonds (Figure 4 and Table 4). Specifically, there is a Nsp3 hybridization of the nitrogen in the C-N-C bond, generating a near bond angle of 120°C that allows the rotation in the C-N bond of the three phenolic groups present in the structure (Figure 4).

### 3.7. Theoretical Physicochemical Properties

We evaluated the anti-inflammatory properties of the TP derivative and evaluated the binding interaction with the putative target enzyme. However, for the prediction of possible drugs, it is necessary to evaluate some physicochemical properties that are important in the design and discovery of new drugs [39]. Therefore, this study determined the preliminary theoretical physicochemical profile of the TP derivative, using quercetin as the positive control (Table 4). The lipophilicity, expressed as the octanol/water partition coefficient (miLogP), and the topological surface area (TPSA) are two markers used to predict the oral bioavailability of new drugs [40–42]. The miLogP results were 1.68 for quercetin and 4.8 for the TP derivative, which correlated with antioxidant capacity (see Table 1). Both compounds possessed LogP values less than 5, which show good water solubility characteristics [39]. The TPSA results showed 131.35 for quercetin and 135.44 for the TP derivative, which were both less than a TPSA value of 150, indicating that the two compounds were valid candidate drugs with good oral bioavailability [39] (Table 4).

## 4. Conclusions

In summary, we have shown that a TP derivative compound previously described (De Hoog et al., 2002) [15] has powerful anti-inflammatory and antioxidant activity because it showed results similar to those described for quercetin, a powerful and known compound with anti-inflammatory and antioxidant properties. Additionally, we observed the interaction of this compound with key residues in the active site of the COX-2 enzyme, an enzyme that is important in the anti-inflammatory response. Finally, regarding the theoretical physicochemical properties of the TP derivative structure, the TP derivative was shown to be a suitable candidate drug with good oral bioavailability (Table 4).

## Figures and Tables

**Table 1:** Antioxidant capacity determined as FRAP and DPPH methods.

Compound	FRAP (*μ*mol Fe^2+^/g)	DPPH (IC_50_, *μ*g/mL)
Quercetin	11,162.5 ± 94.2	1.48 ± 0.1
TP derivative	9,561.1 ± 77.8	4.90 ± 0.16

**Table 2:** Molecular docking analysis for the interaction of COX-2 with the two different ligands.

Ligand	Δ Gbind (kcal mol^−1^)
Quercetin	−54.2 ± 3.6^a^
TP-derivate	−43.6 ± 2.9^b^

Data correspond to the mean ± SE of ten best protein-ligand conformations of five independent docking runs for each ligand with the COX-2. Different superscripted letters indicate significant differences of COX-2 with the different ligands (Tukey HSD test, *p* = 0.05). ΔGbind, total binding energy.

**Table 3:** Interaction analysis between residues of the active site of the COX-2 and the ligands (A) Quercetin and (B) TP derivative.

(A) Quercetin			(B) TP derivative	
Residue of COX-2	Interaction type	% of occupancy	Interaction type	% of occupancy

Lys 518	H-bond (backbone)	86	-	-
Val 214	H-bond (backbone)	61	H-bond (backbone)	99
Arg 362	H-bond (water) (backbone)	67*∗*	-	-
Ser 129	H-bond (water) (backbone)	33*∗*	H-bond (sidechain)	42
Leu 210	H-bond (water) (backbone)	33*∗*	H-bond (backbone)	59
Gln 360	H-bond (sidechain)	86	-	-
Asp 215	-	-	H-bond (water) (sidechain)	98*∗*
Asn 361	-	-	H-bond (water) (backbone)	48*∗*
Asn 361	-	-	H-bond (backbone)	75
Gly 211	-	-	H-bond (water) (backbone)	47*∗*
Phe 128	-	-	Structural role	-

*∗* A water molecule of the form residue-water-ligand mediates the interaction.

**Table 4:** Structural properties evaluation of the two ligands.

Compounds	TPSA	Volume	Nrotb	n-OHNH donors	n-OH acceptors	miLogP
	(Å^2^)	(Å^3^)				
Quercetin	131.35	240.08	1	5	7	1.68
Triazine-Phenol derivative	135.44	347.06	6	6	9	4.80

TPSA: topological polar surface area, nrotb: number of rotatory bonds, n-OHNH: number of hydrogen bond donors, n-OH: number of hydrogen bond acceptors, miLogP: octanol/water partition coefficient. The data was determined using the Molinspiration calculation software v2018.10 [16].

## Data Availability

All data are incorporated in the manuscript.

## Supplementary Materials

Supplementary Materials Figure S1: the result of an analysis of a FT-IR which allows us to characterize our molecule. Figure S2: characterization of our molecule by determining its thermal properties using TGA. Figure S3: an analysis and characterization of mass spectroscopy (MS). Figure S4: the* in silico* analysis of the stability of the protein-ligand complexes (in panel A the TP derivative molecule and the panel B the quercetin molecule used as control). Figure S5: the different residues involved in the interaction with the ligands and shows the percentage of the dynamic time that this interaction was carried out and which type of interaction was carried out.

## References

[1] Nathan C. (2002). Points of control in inflammation. *Nature*.

[2] Tambewagh U. U., Kandhare A. D., Honmore V. S. (2017). Anti-inflammatory and antioxidant potential of Guaianolide isolated from Cyathocline purpurea: Role of COX-2 inhibition. *International Immunopharmacology*.

[3] Rich R. R., Chaplin D. D. (2019). *The Human Immune Response, in Clinical Immunology*.

[4] Masferrer J. L., Zweifel B. S., Manning P. T. (1994). Selective inhibition of inducible cyclooxygenase 2 in vivo is antiinflammatory and nonulcerogenic. *Proceedings of the National Acadamy of Sciences of the United States of America*.

[5] El Sayed M. T., El-Sharief M. A. M. S., Zarie E. S. (2018). Design, synthesis, anti-inflammatory antitumor activities, molecular modeling and molecular dynamics simulations of potential naprosyn® analogs as COX-1 and/or COX-2 inhibitors. *Bioorganic Chemistry*.

[6] Andrade L. N. (2015). Sesquiterpenes from essential oils and anti-inflammatory activity. *Natural Product Communications (NPC)*.

[7] Kim J. H., Kismali G., Gupta S. C. (2018). Natural products for the prevention and treatment of chronic inflammatory diseases: integrating traditional medicine into modern chronic diseases care. *Evidence-Based Complementary and Alternative Medicine*.

[8] Newman D. J., Cragg G. M. (2012). Natural products as sources of new drugs over the 30 years from 1981 to 2010. *Journal of Natural Products*.

[9] Gao Y., Liu J., Chen Y. (2018). Tomato SlAN11 regulates flavonoid biosynthesis and seed dormancy by interaction with bHLH proteins but not with MYB proteins. *Horticulture Research*.

[10] Carvalho A. R., Costa G., Figueirinha A. (2017). Urtica spp.: Phenolic composition, safety, antioxidant and anti-inflammatory activities. *Food Research International*.

[11] Chen L., Teng H., Xie Z. (2018). Modifications of dietary flavonoids towards improved bioactivity: An update on structure–activity relationship. *Critical Reviews in Food Science and Nutrition*.

[12] Garmouna M., Blanchoud H., Teil M.-J., Blanchard M., Chevreuil M. (2003). The new multi-directional polydentate ligand 2,4,6-(di-pyridin-2-yl-amino)-[1,3,5]triazine (dpyatriz) can discriminate between Zn(II) and Co(II) nitrate. *Polyhedron*.

[13] Klenke B., Stewart M., Barrett M. P., Brun R., Gilbert I. H. (2001). Synthesis and biological evaluation of s-triazine substituted polyamines as potential new anti-Trypanosomal drugs. *Journal of Medicinal Chemistry*.

[14] Patel H. S., Patel V. C. (2001). Polyimides containing s-triazine ring. *European Polymer Journal*.

[15] De Hoog P., Gamez P., Driessen W. L., Reedijk J. (2002). New polydentate and polynucleating N-donor ligands from amines and 2,4,6-trichloro-1,3,5-triazine. *Tetrahedron Letters*.

[16] Cheminformatics Bratislava. http://www.molinspiration.com/services/properties.html.

[17] Duncan R., Izzo L. (2005). Dendrimer biocompatibility and toxicity. *Advanced Drug Delivery Reviews*.

[18] Castro R. I., Forero-Doria O., Soto-Cerda L., Peña-Neira A., Guzmán L. (2018). Protective effect of pitao (Pitavia punctata (R. & P.) Molina) polyphenols against the red blood cells lipoperoxidation and the in vitro ldl oxidation. *Evidence-Based Complementary and Alternative Medicine*.

[19] Forero-Doria O., Astudillo L., Castro R. I. (2014). Antioxidant activity of bioactive extracts obtained from rhizomes of cyperus digitatus roxb. *Boletin Latinoamericano y del Caribe de Plantas Medicinales y Aromaticas*.

[20] (2015). *Release, S., 4: LigPrep, version 3.6*.

[21] Shelley J. C., Cholleti A., Frye L. L., Greenwood J. R., Timlin M. R., Uchimaya M. (2007). Epik: a software program for pK_a_ prediction and protonation state generation for drug-like molecules. *Journal of Computer-Aided Molecular Design*.

[22] Jorgensen W. L., Maxwell D. S., Tirado-Rives J. (1996). Development and testing of the OPLS all-atom force field on conformational energetics and properties of organic liquids. *Journal of the American Chemical Society*.

[23] Friesner R. A., Banks J. L., Murphy R. B. (2004). Glide: a new approach for rapid, accurate docking and scoring. 1. Method and assessment of docking accuracy. *Journal of Medicinal Chemistry*.

[24] Schrödinger Release 2016-4: Desmond Molecular Dynamics System, D. E. Shaw Research, New York, NY, 2016.

[25] Shivakumar D., Williams J., Wu Y., Damm W., Shelley J., Sherman W. (2010). Prediction of absolute solvation free energies using molecular dynamics free energy perturbation and the opls force field. *Journal of Chemical Theory and Computation*.

[26] Noparvar-Qarebagh A., Roghani-Mamaqani H., Salami-Kalajahi M. (2016). Novolac phenolic resin and graphene aerogel organic-inorganic nanohybrids: High carbon yields by resin modification and its incorporation into aerogel network. *Polymer Degradation and Stability*.

[27] Pan D. C., Myerson J. W., Brenner J. S. (2018). Nanoparticle properties modulate their attachment and effect on carrier red blood cells. *Scientific Reports*.

[28] Li Y., Yao J., Han C. (2016). Quercetin, inflammation and immunity. *Nutrients*.

[29] Langley-Evans S. C. (2000). Antioxidant potential of green and black tea determined using the ferric reducing power (FRAP) assay. *International Journal of Food Sciences and Nutrition*.

[30] Borneo R., León A. E., Aguirre A., Ribotta P., Cantero J. J. (2009). Antioxidant capacity of medicinal plants from the province of Córdoba (Argentina) and their *in vitro* testing in a model food system. *Food Chemistry*.

[31] Cao G., Sofic E., Prior R. L. (1997). Antioxidant and prooxidant behavior of flavonoids: structure-activity relationships. *Free Radical Biology & Medicine*.

[32] Nagayama M., Niwa K., Nagayama T., Ross M. E., Iadecola C. (1999). The cyclooxygenase-2 inhibitor NS-398 ameliorates ischemic brain injury in wild-type mice but not in mice with deletion of the inducible nitric oxide synthase gene. *Journal of Cerebral Blood Flow & Metabolism*.

[33] Grosser T., Fries S., FitzGerald G. A. (2006). Biological basis for the cardiovascular consequences of COX-2 inhibition: therapeutic challenges and opportunities. *The Journal of Clinical Investigation*.

[34] Aid S., Langenbach R., Bosetti F. (2008). Neuroinflammatory response to lipopolysaccharide is exacerbated in mice genetically deficient in cyclooxygenase-2. *Journal of Neuroinflammation*.

[35] Aid S., Silva A, Candelario-Jalil E., Choi S. H., Rosenberg G. A., Bosetti F. (2010). Cyclooxygenase-1 and-2 differentially modulate lipopolysaccharide-induced blood–brain barrier disruption through matrix metalloproteinase activity. *Journal of Cerebral Blood Flow & Metabolism*.

[36] Choi S.-H., Langenbach R., Bosetti F. (2008). Genetic deletion or pharmacological inhibition of cyclooxygenase-1 attenuate lipopolysaccharide-induced inflammatory response and brain injury. *The FASEB Journal*.

[37] Choi S.-H., Aid S., Choi U., Bosetti F. (2010). Cyclooxygenases-1 and-2 differentially modulate leukocyte recruitment into the inflamed brain. *The Pharmacogenomics Journal*.

[38] Falkovskaia E., Sengupta P. K., Kasha M. (1998). Photophysical induction of dual fluorescence of quercetin and related hydroxyflavones upon intermolecular H-bonding to solvent matrix. *Chemical Physics Letters*.

[39] Rengasamy K. R. R., Slavětínská L. P., Kulkarni M. G., Stirk W. A., Van Staden J. (2017). Cuparane sesquiterpenes from Laurencia natalensis Kylin as inhibitors of alpha-glucosidase, dipeptidyl peptidase IV and xanthine oxidase. *Algal Research*.

[40] Clark D. E. (1999). Rapid calculation of polar molecular surface area and its application to the prediction of transport phenomena. 1. Prediction of intestinal absorption. *Journal of Pharmaceutical Sciences*.

[41] Clark D. E. (1999). Rapid calculation of polar molecular surface area and its application to the prediction of transport phenomena. 2. Prediction of blood-brain barrier penetration. *Journal of Pharmaceutical Sciences*.

[42] Chang L. C. W., Spanjersberg R. F., Von Frijtag Drabbe Künzel J. K. (2004). 2,4,6-Trisubstituted pyrimidines as a new class of selective adenosine A1 receptor antagonists. *Journal of Medicinal Chemistry*.

