# Supplementary material for: In Silico and In Vitro Analysis of the 4,4′,4′′-((1,3,5-Triazine-2,4,6-triyl)tris(azanediyl))triphenol), an Antioxidant Agent with a Possible Anti-Inflammatory Function
Source: Biomed Res Int. 2019 May 22;2019:9165648. doi: 10.1155/2019/9165648 (PMC6556288; doi:10.1155/2019/9165648)
Supplement: Supplementary Materials — Figure S1: the result of an analysis of a FT-IR which allows us to characterize our molecule. Figure S2: characterization of our molecule by determining its thermal properties using TGA. Figure S3: an analysis and characterization of mass spectroscopy (MS). Figure S4: the in silico analysis of the stability of the protein-ligand complexes (in panel A the TP derivative molecule and the panel B the quercetin molecule used as control). Figure S5: the different residues involved in the interaction with the ligands and shows the percentage of the dynamic time that this interaction was carried out and which type of interaction was carried out. [file 9165648.f1.pdf]

Supplementary Information

In-silico **and** ***in vitro*** **analysis of the 4,4',4''-((1,3,5-triazine-2,4,6-triyl)tris(azanediyl))triphenol), an antioxidant agent with a possible anti-inflammatory function**

Ricardo I. Castro, Felipe Valenzuela-Riffo, Luis Morales-Quintana

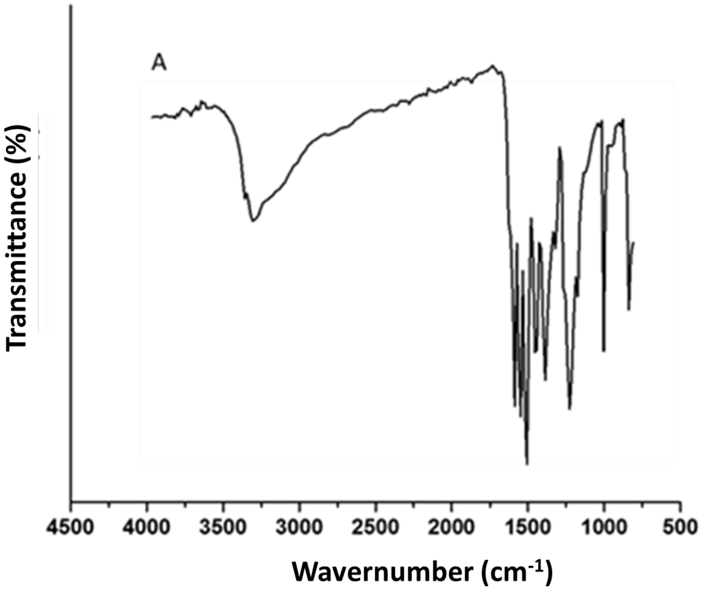

Figure S1. FTIR spectrum of synthetic TP derivate

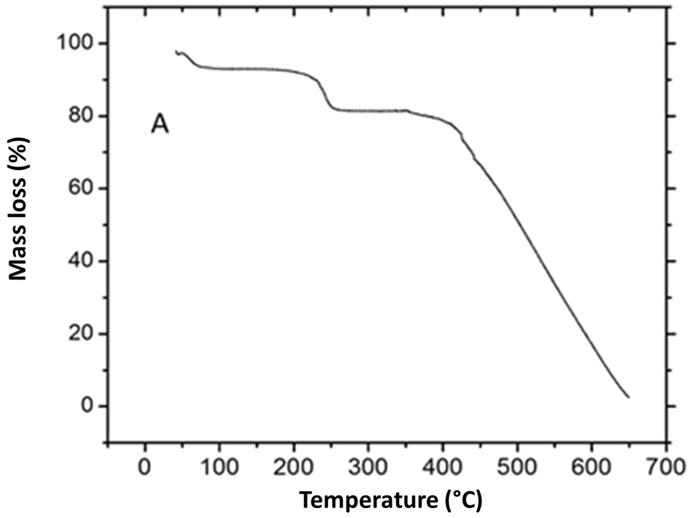

Figure S2. TGA thermogram of triazine phenol derivative.

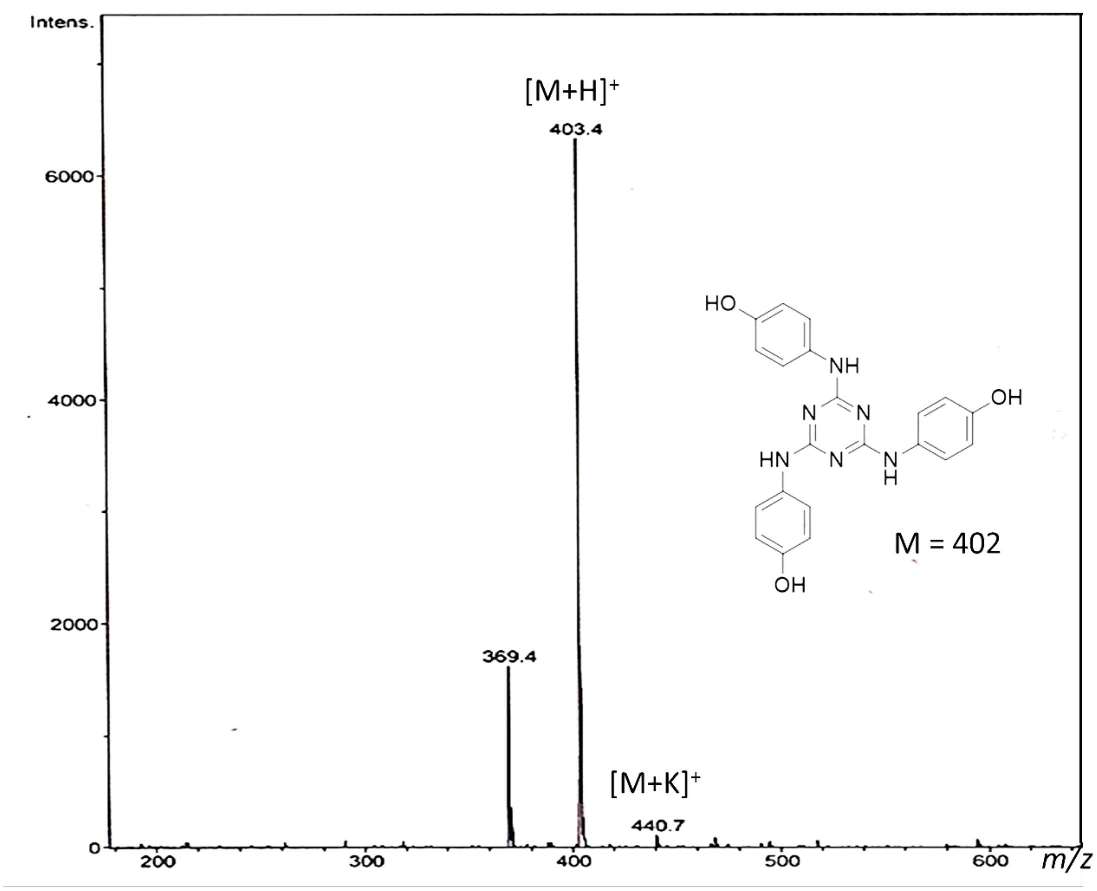

Figure S3. MASS spectrometry spectrum.

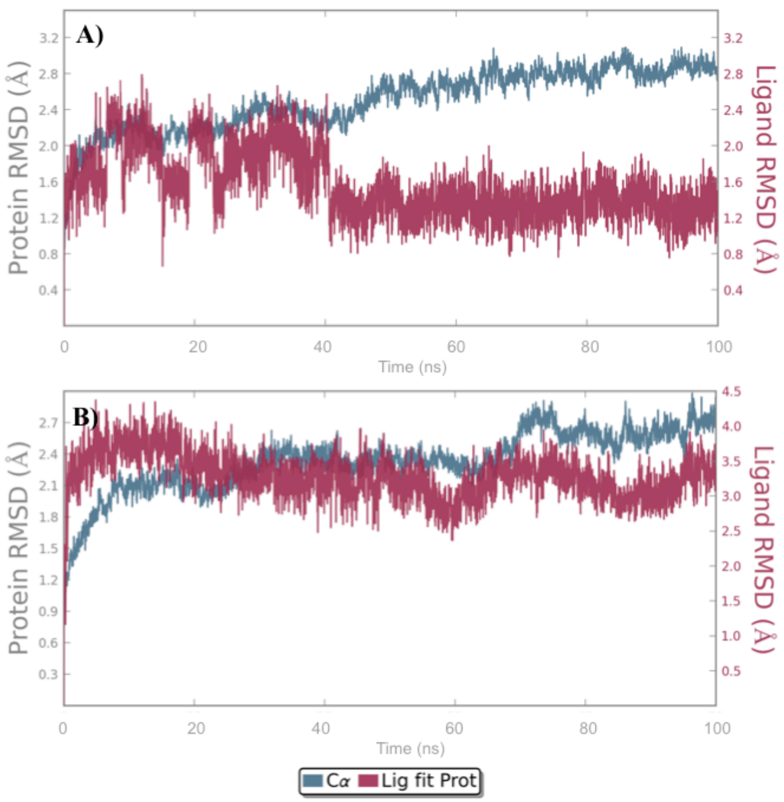

Figure S4. RMSD of the protein ligand complex. A) COX-2_TP derivative complex. B) COX-2_Quercetin complex. In dark blue showed the RMSD value of the Cα of the COX-2 protein, in red showed the RMSD value of the each ligand in the respective graph.

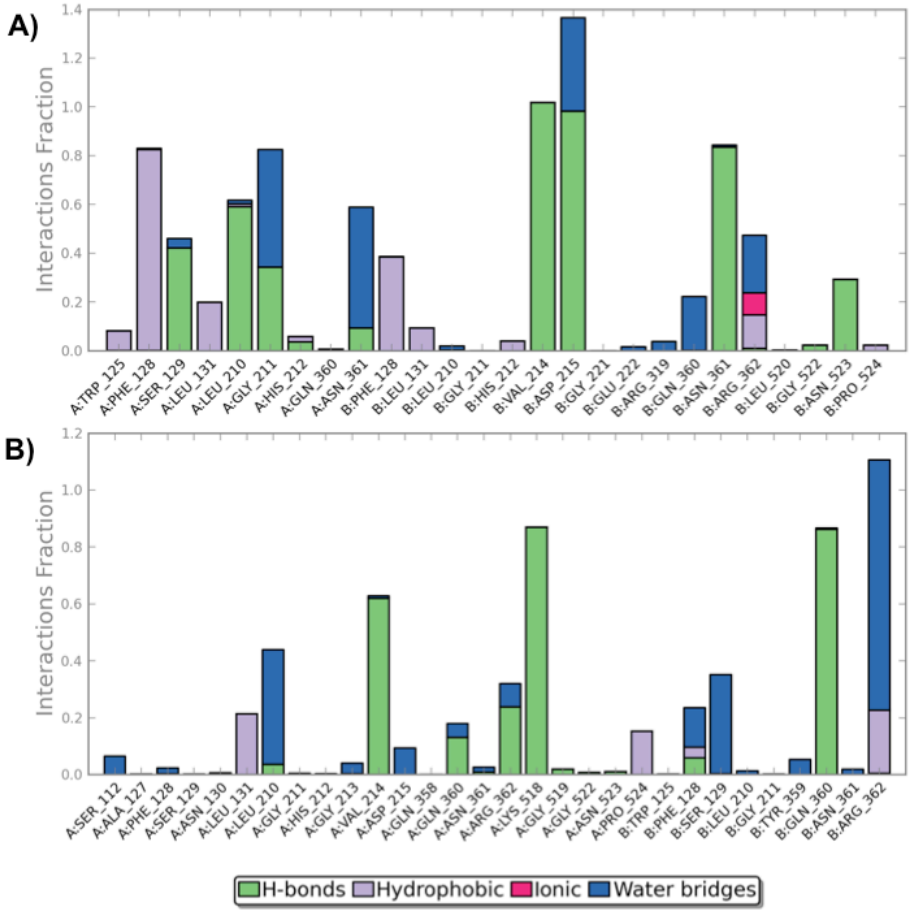

Figure S5. Protein-ligand interactions (or 'contacts') in each protein-ligand complex.
A) COX-2_TP derivative complex. B) COX-2_Quercetin complex. Four types of interaction were described; the hydrogen bonds are showed in green, the hydrophobic interactions are showed in purple, the ionic interactions are showed in red and water bridges is showed in blue.
